# Challenges to manage pandemic of coronavirus disease (COVID-19) in Iran with a special situation: a qualitative multi-method study

**DOI:** 10.1186/s12889-021-11973-5

**Published:** 2021-10-22

**Authors:** Hamidreza Khankeh, Mehrdad Farrokhi, Juliet Roudini, Negar Pourvakhshoori, Shokoufeh Ahmadi, Masoumeh Abbasabadi-Arab, Nader Majidi Bajerge, Babak Farzinnia, Pirhossain Kolivand, Vahid Delshad, Mohammad Saeed Khanjani, Sadegh Ahmadi-Mazhin, Ali Sadeghi-Moghaddam, Saiedeh Bahrampouri, Ulrich Sack, Marcus Stueck, Bernd Domres

**Affiliations:** 1 Health in Emergency and Disaster Research Center, the University of Social Welfare and Rehabilitation Sciences, Tehran, Iran; 2 Department of Nursing, School of Nursing and Midwifery, Guilan University of Medical Sciences, Rasht, Iran; 3 National Emergency Medical Organization, Ministry of Health and Medical Education, Tehran, Iran; 4 School of Public Health, Qom University of Medical Sciences, Qom, Iran; 5 Department of Counseling, University of Social Welfare and Rehabilitation Sciences, Tehran, Iran; 6 Department of Nursing, Dezful University of Medical Sciences, Dezful, Iran; 7 Institute of Clinical Immunology, Medical Faculty, University of Leipzig, Leipzig, DE Germany; 8 DPFA Academy of Work and Health, Leipzig, Germany; 9 Surgery Department, University of Tubingen, Tübingen, Germany

**Keywords:** COVID-19, Iran, Multi-method study, Challenge, Lessons learned

## Abstract

**Background:**

With the unprecedented expansion of COVID-19 in the world since December 2019, Iran’s health system, like other countries, faced various challenges in managing the disease, which led to numerous experiences and lessons learned. This study was conducted to identify these challenges regarding unique political, economic, and cultural issues, which could help other countries with similar situations.

**Methods:**

The present study was performed using a qualitative multi-method approach with a content analysis method. The data were collected through in-depth and semi-structured interviews and focused group discussions with 60 key persons who were selected purposefully, including policymakers, health care workers, and affected people by the disease, and the review of all available national reports between February 21, 2020, and March 22, 2021. The data collection and analysis were done simultaneously.

**Results:**

Identified critical challenges for the management of COVID-19 in the health system were limited evidence and scientific controversies, poor social prevention and social inequalities, burnout and sustained workload among healthcare workers, improper management of resources and equipment, the lack of a guideline for contact tracing, and patient flow management, and mental health problems in the community.

**Conclusions:**

According to our results, measures should be taken to conduct a continuous comprehensive risk assessment and develop a national response plan with an emphasis on precise contact tracing, active screening, patient flow, paying attention to the psychological and social dimensions of the disease, and also transparency of social inequalities in the face of risk factors of the COVID-19. Also, the social protection programs should become a vital tool for policymakers and supporting the vulnerable groups using the capacity of the community and international cooperation to develop a vaccine, which is difficult to procure due to the sanctions.

## Background

The pandemic of coronavirus disease 2019 (COVID-19) has led to a high rate of morbidity and mortality and imposed a burden of billions of dollars [1]. How each country responds to the pandemic in the coming weeks and months will be critical for affecting the disease trajectory and having a coordinated international pandemic response.

The rapid spread of the disease with several waves and peaks for around a year and a half has imposed a lot of pressure on the health system and the other parts of the community worldwide. Many health care professionals are on the front lines of battling the COVID-19 public health crisis, and many hospitals are overwhelmed with suspected or infected cases of COVID-19. This global pandemic can be characterized by the complexity of its origin, the speed of its spread, and the unpredictability of its scale and impact [2]. Quarantine and isolation measures immediately implemented to control the pandemic might potentially have negative psychological and social effects, specifically vulnerable people, such as frontline medical workers, children, and older adults [3, 4]. The COVID-19 crisis emphasizes the connections between environmental changes and the emergence of infectious diseases and notifies us about the serious need to prevent such pandemics, as their control can be extremely difficult in a globalized world. Several mental and social disorders caused by pandemics or epidemics can affect people’s daily activity. A fear of becoming infected or death makes the condition worse and people experience stress and anxiety to some extent, and psychological disorders become common [5]. In a study conducted during the initial phase of the coronavirus outbreak, more than half of the respondents rated the psychological impact as moderate-to-severe, and about one-third reported moderate-to-severe anxiety [6].

There is still significant ambiguity as the pandemic continues to evolve. COVID-19 vaccines have started to be rolled out in countries worldwide, but this does not mean the crisis is close to being solved or people can return to normal life [7]. We are relocating to a new phase of the pandemic, and it is expected that the world will be involved in making decisions about the COVID-19 issues and consequences issues for several years. There is no immediate solution offered at present [7]. People worldwide must be prepared to cope with both the demands and the consequences of living with masks, physical distancing, and hand hygiene for a long period [8].

On February 19, 2020, Iran confirmed two patients that were infected with the COVID-19 in Qom city, and more cases were reported in Tehran in a few days later [9]. Iran is the third country with the highest number of reported COVID-19 cases after China, and Italy, up to March 16, 2020, being the first in the Middle East region and perhaps becoming a primary source of imported cases in this area [10]. The spread of the disease in Iran, like in China, coincided with the Iranian New Year holidays and the relocation of populations from virus-infected cities, such as Tehran and Qom to other parts of the country. Therefore, the government imposed some restrictions, like travel banning and closure of the schools and universities [9]. With the occurrence of COVID-19, the Iranian health system faced various challenges in managing the disease with several peaks, which were exacerbated by multiple severe sanctions. Accordingly, the government decided to reopen too early, which led to an increase in the number of confirmed cases and deaths from May 2, 2020, and experiencing some of the worst scenarios from September until the end of December 2020 and March until May 2021. Therefore, exploring the experiences from challenges in Iran, with its unique fluctuation in the number of infected cases and deaths can help to effectively manage the pandemic and reduce its burden on health managers inside the country and abroad. Therefore, this comprehensive study was conducted for the first time in Iran to explore the challenges for managing the COVID-19 pandemic in Iran, regarding its unique political, economic, and cultural issues, which can be helpful for other countries with a similar situation.

## Methods

The present study was carried out with a multi-method approach using qualitative content analysis to explore a deeper understanding of the experiences gained from managing COVID-19 in Iran. The study was started with a focus group discussion (FGD.) with 60 key persons in five groups of people with different experiences and backgrounds from various departments to explore the health system’s challenges to manage COVID-19 in Iran. These FGDs were conducted at two intervals of 3 months, started in August 2020.

Following FGDs and to develop the explored concepts, in-depth individual interviews were conducted. It is noteworthy that according to the rules of social distance at the time of the COVID-19 outbreak, the interviews were conducted by telephone or via Skype. A total of 60 people participated in FGDs, of whom 30 cases were recruited for in-depth interviews. The study participants were selected purposefully and sampling was continued until saturation (Continuation of sampling until no new relevant data were generated.

Moreover, all the available daily national documents and reports related to the management of COVID-19 were collected by searching the Internet and the Ministry of Health and Medical Education (MoHME) between February 21, 2020, and March 22, 2021.

Simultaneously with data collection, qualitative content analysis was performed through the Graneheim and Lundman approach. In addition to the content of the selected documents and reports, the text of the FGDs and interviews was also transcribed and then, read several times, and the key points and concepts related to the objectives of the research were identified. Data were analyzed concurrently with data collection using content analysis. Interview transcripts were read to grasp participants’ perceptions and then, the meaning units were identified and coded with conceptual labels. Preliminary coding was performed and then, the codes were permanently compared in terms of similarity and difference. After this stage, each set of similar codes developed sub-categories and categories, and finally, challenges were explored [11–14].

### Rigor study power

This study has several strengths. The information was collected and analyzed since the first time the country formed the Coronavirus Headquarters (February 21, 2020). Also, to improve the study power, the multi-method approach, including individual interviews, FGDs, and document analysis, was used.

To evaluate the trustworthiness of the findings, Guba and Lincoln’s criteria, including credibility, transferability, dependability, and confirmability were applied [15]. The researchers were involved in the data collection until reaching data saturation. The research team members reviewed the data independently and then, coded, categorized, and finally, compared them with each other. A summary of the preliminary results was given to the participants for matching the results with their experiences and documenting all stages of the research to make it usable for other researchers.

## Results

To explore the challenges to manage COVID-19 in Iran, 60 people from different disciplines were studied (Table 1).

**Table 1:** Demographic characteristics of participants

Demographic characteristics	Sub-Category	Number (% of the sample)
**Gender**	Male	**36 (60)**
	Female	**24 (40)**
**Age (years)**	25–35	**2 (3.3)**
	36–45	**17 (28.3)**
	≥46	**41 (68.3)**
**Participant’s status**	Managers of the Ministry of Health (top managers)	**4 (6.6)**
	University Administrators	**6 (10)**
	Physician (infectious disease, emergency medicine, pulmonary disease specialist, and general practitioner)	**10 (16.6)**
	Health care worker (nurse, psychologist, health practitioner, and social worker)	**25 (41.6)**
	Patients and their affected family members	**10 (16.6)**
	Suspected cases (unconfirmed cases of COVID-19)	**5 (8.3)**

The explored challenges were classified into six main categories: the limited evidence and scientific controversies, inadequate attention to the political, social, cultural, and economic issues and community involvement, burnout and sustained workload of health care workers, improper management of the resources and equipment, lack of updated and agreed guidelines for contact tracing and patient flow, and long-lasting community mental health problem and shortcomings in supporting vulnerable groups by the society (Table 2).

**Table 2:** The main categories of explored challenges and Statements of the interviewees

Findings	main categories of explored challenges	Statements of the interviewees
Category 1	**The limited evidence and scientific controversies**	“Given that the virus is very new and there is no accurate experience or information about it, for For example, in the case of reinfection, I don’t think it can be proven that whether it is reinfection or a recurrence of the disease.”
Category 2	**Inadequate social prevention and social inequalities**	“Some people have to work every day with low income. They can’t follow social distancing rules”.
Category 3	**Burnout and sustained workload among healthcare workers**	“In the early days, since the doctors themselves were not very familiar with the disease or there were no screening tests …, and it was the beginning of the work, the patients came to stay [at the hospital] for 3–4 days, and we used to do their treatment and care with ordinary clothes and a simple mask. In fact, we considered them as a regular patient. A few days later, we would find out that the case is infected with Coronavirus; that put a lot of pressure on us and increased stress. We were terrified of all patients, and we took care of each of them with horror.” “I do not know if this is the second wave or not, but the number of patients has been sharply increased, and again, we are shocked.”
Category 4	**Improper management of the resources and equipment**	“I can say that for the first two weeks, the staff was in a lot of trouble. Gloves, masks, and alcohol were not there. These were not available in the market at all. “ “Different and sometimes contradicted protocols make hospital staff confused about how they can protect themselves.”
Category 5	**Lack of** updated **and agreed guidelines for contact tracing and patient flow**	“If someone suspected to have the disease, there is no trustable and available sources to help him or her. Even Health care workers are completely confused as to where to go and what should they do”
Category 6	**Community mental health problems**	“… everyone gets depressed. Social relationships are limited. People are afraid of one another. You feel like you are the center of infection. […] It’s psychologically problematic. For example, I have become very aggressive. I get angry very quickly and severely. I’m a little bored.”

### The limited evidence and scientific controversies

There is limited scientific evidence nationally and internationally documented and agreed about the clinical manifestations, treatment, and course of the disease. People’s reactions to the disease appear to be divergent, resulting in different clinical manifestations, diverse and changing clinical course, and different prognoses. On the other hand, there is currently limited evidence available on how the disease is developed and how the disease is spread. Due to the inability to analyze the factors affecting the disease process and the inability to manage other accompanying and underlying disorders, vulnerable groups cannot be identified and supported with certainty.

### Poor social prevention and social inequalities: inadequate attention to political, social, cultural, and economic issues and community participation

Weakness in social prevention was a shortcoming in preventive measures of the COVID-19, which was followed by the weakness in risk communication, weakness in community participation, poor economic situation and livelihood issues, and a lack of proper inequality in the face of the disease and access to resources, and integrated and enforcement rules and regulations for social distancing. People are informed and overwhelmed with multiple media in the country 24 h/7 days. Due to the late notification of the spread of the disease in the country and the lack of transparency, the ground for reducing people’s trust was provided, which caused inadequate social prevention in the community.

### Burnout and sustained workload among health care workers

This challenge included constant psychological pressures, lack of adequate support, inability to manage human resources, and neglect of their own needs by instilling a sense of heroism. The health system has experienced a high workload, a reduction in mental strength, and physical, emotional, and psychological fatigue of health care workers due to long-term working hours and increasing the number of hospitalized patients again and again several times, in particular, since September 2020.

Limited knowledge about the nature of the disease, the possibility of transmitting to the staff due to late diagnosis of patients, asymptomatic and pre-symptomatic infected patients and staff, and finally, being worried about the possibility of transmitting the disease to their family makes health care workers nervous. Moreover, being a witness of many patients and the high mortality rate and the lack of effective treatment for this disease had created stressful conditions for them. In addition to the constant psychological pressure on employees, they were not adequately supported. The statistics showed that the country was at the forefront of the infection and death of hospital staff. Besides, in the first few weeks, there was no proper protocol to protect infected employees and re-employ them, and even some employees did not have job security, and they were afraid of losing their job. Furthermore, the general shortage of staff in the health system, the departure of many staff during the crisis, and increasing the number of hospitalized patients had led to a massive workload on staff. It is evident that increasing the number of infected and hospitalized patients has recently frustrated hospital staff and even manages more.

### Improper management of the resources and equipment

Despite the need for resources and equipment in some areas, request management, strategic supply chain, and distribution were not well managed; therefore, some hospitals and staff did not have enough resources. Besides, it can be said that sanctions, as a political-economic factor, more than any other aspect, have challenged Iran’s ability to provide needed supply and medical equipment. Being unable to procure vaccines because of the sanction puts a lot of pressure on the health system.

### Lack of updated and agreed guidelines for contact tracing and patient flow

There was no proper protocol for screening, contact tracing, referral, patient follow-up, call centers, admission, treatment, temporary health centers, and rehabilitation of the patients. This led to the confusion of the patients and the health care system and a lack of adequate and effective care for people in need during the first few weeks.

### Community mental health problems: long-lasting community mental health problems and shortcomings in support of vulnerable groups by the society

The occurrence of COVID-19 and its management measures were accompanied by many changes in the lifestyle of healthy people, patients, and their families, which in general, has increased personal tensions in society. In some people, the unknown nature of the disease, fragile economy, the obscure process of the diagnosis and treatment, and receiving too much information about the disease, caused by changes in business and living conditions, social interaction, facing anxiety and fear, and living in uncertainty, has changed the people’s lifestyle. Besides, focusing on treatment and hospital surge capacity and ignoring non-pharmaceutical interventions have increased the number of deaths in older adults and other vulnerable groups with underlying medical conditions, such as cardiovascular disease, diabetes, chronic respiratory disease, and cancer.

## Discussion

At the beginning of the COVID-19 outbreak in Iran, adequate measures were recommended and taken by the health system to control this epidemic. These interventions can be classified into three main parts: 1) Measures to change the health behaviors of the community and improve self-care; 2) Social distance plans; and 3) Active screening, finding patients, and isolating COVID-19 cases, which were not wholly fulfilled.

These interventions caused Iran to have acceptable success in the relative control of the first wave of the COVID-19 for several weeks. Our results showed that despite the efforts made, due to the limited evidence and scientific controversies, severe sanctions, the insufficient preparedness of society and the health system, lack of a plan for risk communication and community engagement, and after an early reopening, challenges arose in the management of the disease and the number of deaths and hospitalized people has increased for several times. The challenges explored by the study were classified into six main categories: the limited evidence and scientific controversies, inadequate attention to political, social, cultural, and economic issues and community involvement, burnout and sustained workload of health care workers, improper management of the resources and equipment, lack of updated and agreed guidelines for contact tracing and patient flow, and long-lasting community mental health problems and shortcomings in the support of vulnerable groups by the society.

The nature of the COVID-19 and the way(s) it had spread globally are still unknown, which has led to the rapid spread of the disease in different countries. The transmission potential in asymptomatic, symptomatic, mild, and severe cases remains unknown. In some cases of COVID-19, we face inconsistencies between laboratory diagnosis and clinical and radiographic findings. In line with this part of the results, some studies have focused on the unknown nature of the virus and its complex etiologies [16, 17].

In the early onset of a pandemic, there were discrepancies in the recommendations from health authorities, such as the World Health Organization (WHO) and Centers for Disease Control and Prevention (CDC) [18]. The understanding of human, environmental, and animal contributions to the pandemic was poor. Several methods and medications have been recommended for the treatment of the disease [19–21]. In line with this study, in the American community, scientific uncertainty has been an influential factor in adherence to preventive measures by the public population [22]. Contradictions in scientific evidence and media news have also confused health care providers, and as a challenging issue, it has caused people to distrust health advice. Scientific uncertainty about the COVID-19 pandemic has caused ambiguity aversion, cognitive bias, and emotional reactions in the community [23].

The political, social, cultural, and economic status of society was recognized as an influential factor. Social and religious norms, especially in traditional societies, affect the level of exposure of people. The existence of religious and traditional ceremonies that are accompanied by crowds has been an influential factor, and these aggregations have made social distancing measures more difficult [24, 25]. The lack of sufficient financial support and national protection program from the Iranian government to the people during quarantine have made it impossible for most of the people to comply with quarantine and continue their business at the community level. This has made it impossible to control the disease because social distancing and reduced exposure, which are essential principles of reducing the risk of the disease, have become virtually impossible [25].

In some cases, some people due to poor economic conditions have not been able to provide disinfectants and masks, making it impossible to break the chain of infection. During the pandemic, different countries, based on their cultural needs and financial ability, have used different strategies and programs to control the disease. In India, the announcement of a nationwide home quarantine for 3 weeks from March 26, 2020, and the help of police forces to prevent people from leaving their homes, closing cultural and religious centers, stopping the tourism industry, and holding some popular Indian sports, such as cricket without spectators were some actions to control the disease [26]. Egypt also closed its borders and canceled international flights until April 23, 2020, closed schools, universities, sports clubs, restaurants, and cultural venues, suspended cultural and artistic activities, mosques and churches, banned collective activities, and the holding of Iftar ceremonies during the month of Ramadan, and released eligible prisoners to prevent the spread of the disease in the country [27]. The implementation of the quarantine program and its extension until the control of the disease, the performance of the social distance plan, home-to-home screening of people by health personnel, and the prohibition of public gatherings were other programs of this country [28]. The extent and quality of implementing such control programs; however, depend entirely on factors, such as the economic status of the governments and the extent, to which they support businesses affected by the disease [29, 30].

The intensive work of health care providers during COVID-19 physically and emotionally has made them exhausted [31]. Adequate, trained, experienced, responsible, and accountable staff plays an essential role in achieving the health system’s goals. A recent study in Iran showed that in terms of the subscale score of NASA-TLX, nurses obtained higher scores in mental pressure, physical pressure, time pressure (temporal), and frustration compared to other jobs. Moreover, nurses had significantly more workloads compared to other jobs [32]. Health care workers need to be motivated to provide quality services to the community. Sometimes, due to the lack of evidence and unrealistic self-confidence, health care workers neglect proper personal protective equipment (PPE), which should be monitored and supported by the health system. It is necessary to prepare a database of employees of the health system, retirees, and volunteers and organize them in alternative relief teams.

In this regard, Brazil has listed the benefits of official employees and employers from the short-term work programs supported by the government and unemployment insurance and payment for the first 15 days of sick leave to the employees who have tested positive for COVID-19. India has also allocated medical insurance for health care providers [33]. In Germany, DBfK – Bundesverband has a cooperation with the Federal Chamber of Psychotherapists to provide telephone counseling services to nurses free of charge. In Taiwan, nurses who have taken care of suspected or confirmed cases of COVID-19 may are provided with three and 14 days of paid leave, respectively [34].

Improper management of resources and equipment was found to be one of the most effective barriers to control COVID-19 in this study. Several studies have also shown the importance of supply chain impact and resource scarcity [35–37].

Equipment, such as PPE, ventilators, oxygen, and diagnostic kits should be available to control the disease [38]. Providing the necessary equipment in an emergency by making changes to upstream rules, developing guidelines, and transparent processes for the strategic supply chain is essential to deal with COVID19. Preventing the spread of infection at the community level and among health care providers relies on the use of PPE. This equipment should be readily available to the public to maximize its efficiency. Sometimes disruption in the supply chain of equipment used by people and health centers has led to the spread of the disease and consequently, increased demand, and eventually, this defective cycle has led to more shortage of equipment and further spread of the disease [36, 39, 40].

Contact tracing is an essential control measure to break the chains of COVID-19 transmission [41]. Because COVID-19 is highly variable and is asymptomatic in many nonspecific individuals, there is a need to advance the contact tracing to prevent further transmission [42]. Action based on a coherent protocol and guideline that includes all stages of screening, contact tracing, patient follow-up, treatment, and rehabilitation of patients is a fundamental principle in the comprehensive control and management of the pandemic. of the agreed guidelines for contact tracing and patient flow were not comprehensive [43–45]. The lack of an integrated and updated contact tracking system in Iran sometimes has caused an asymptomatic person to be left in the community, despite a positive test result. In some cases, family or co-workers are unaware that the person is a carrier. Contact tracking in China has been a fundamental principle in disease control [46, 47]. Covid-19 pandemic has been associated with countless stressors [48]. In low-income economies or those with no adequate government support, these tensions were excessive. The emergence of mental health problems was one of the main manifestations of these stressors. Depressive and anxiety disorders are the most common disorders seen during the pandemic period [49–51]. During the COVID-19 pandemic in brazil, anxiety and depressive symptoms increased 7.4 and 6.6 times, respectively [52]. Social inequality in Iran has led to more health and economic consequences for people during the COVID-19 outbreak [25, 53, 54]. People with poor economic status are more likely to experience the devastating consequences of the COVID-19 pandemic compared to others [55].

Finally, to manage the next wave of the disease, it is recommended to make a clear decision on when to reopen educational centers, monitoring the implementation of social distancing protocols, establishing more strict and serious rules for traffic restrictions, quarantine of contacted and vulnerable people, real-time exchange of information with establishing a trustable communication with the people to educate and accompany them for quarantine and social distancing. Providing appropriate and timely social support for vulnerable groups can decrease the number of patients and deaths. Supporting the country to procure vaccines at least for vulnerable groups by the international community can help the health system to manage the next coming waves or peaks.

### Limitations

Some limitations should be considered when interpreting the results of this study. The main limitation of this study like other qualitative studies is the lack of generalizability of the results, which is related to the nature and philosophical assumptions of the qualitative paradigm, which our study is not an exception. Because of the pandemic, it was not possible to do face-to-face interviews, which could help to explore more in-depth experiences of the study participants and probe unclear points of view. Moreover, some health care workers and policymakers were reluctant to participate in the study and share their experiences, and finally, some related reports were not available for analysis. To overcome these limitations, we tried to use the triangulation approach for data gathering, including FGD, in-depth individual interviews and document analysis, and selecting participants with maximum diversity of experiences.

## Conclusion

The results of this study indicated that considering the scientific controversies and using trustworthy evidence, paying attention to the political, social, cultural, and economic situation, burnout and sustained workload of health care workers, effective management of the resources and equipment, developing updated and agreed guidelines for contact tracing and patient flow, and planning for mental health in the community, and national social protection program are crucial factors to manage COVID-19. Assessing these challenges in the health system in response to COVID-19 can improve the knowledge of service providers and can be served as a primary framework for planning by the health system to enhance readiness to manage this long-lasting and devastating event and similar situations. This study indicated the need for public participation in the whole process of disease management, paying attention to social inequality in the face of the disease, and the need to address various aspects of the disease to deal with the disease. Continuous risk assessment, predicting possible scenarios for similar conditions, and developing a comprehensive national response plan are also recommended. Longitudinal studies are needed to fully understand the symptoms, the time of the pathogenesis, and the long-term effects of the disease on health and psychological, social, and other dimensions. Moreover, transparency of social inequalities faces risk factors so that sometimes economic resources are not eligible enough to provide adequate support to different groups in the society, especially vulnerable people. In Iran, extensive sanctions and insufficient resources have reduced the access of vulnerable groups to the vaccine, needed resources, and services. We believe that the complete transfer of disease management to the health sector has sometimes led to a lack of attention to other influential aspects of the disease, such as economic, social, and cultural dimensions. Countries that reopened early without a reduction in transmission rate are suffering from new spikes of contamination. However, countries that quarantined quickly and rigorously could control the virus more effectively. Finally, a strong risk communication strategy should remind citizens that the pandemic is not over, and also international collaboration is critical for manage this devastating pandemic.

## Data Availability

The data that support the findings of this study are available from Mehrdad Farrokhi, but restrictions apply to the availability of these data, which were used under license for the current study, and so are not publicly available. Data are however available from the authors upon reasonable request and with the permission of Mehrdad Farrokhi.
